# Insights into the role and regulation of TCTP in skeletal muscle

**DOI:** 10.18632/oncotarget.13009

**Published:** 2016-11-01

**Authors:** Craig A. Goodman, Allison M. Coenen, John W. Frey, Jae-Sung You, Robert G. Barker, Barnaby P. Frankish, Robyn M. Murphy, Troy A. Hornberger

**Affiliations:** ^1^ Department of Comparative Biosciences, School of Veterinary Medicine, University of Wisconsin-Madison, Madison, Wisconsin, 53706, USA; ^2^ Centre for Chronic Disease Prevention and Management, College of Health and Biomedicine, Victoria University, Melbourne, Victoria, 8001, Australia; ^3^ Institute for Sport, Exercise and Active Living (ISEAL), Victoria University, Melbourne, Victoria, 8001, Australia; ^4^ Department of Biochemistry and Genetics, La Trobe Institute for Molecular Science, La Trobe University, Melbourne, Victoria, 3086, Australia

**Keywords:** muscle hypertrophy, muscle atrophy, cancer, duchenne muscular dystrophy, proteostasis

## Abstract

The translationally controlled tumor protein (TCTP) is upregulated in a range of cancer cell types, in part, by the activation of the mechanistic target of rapamycin (mTOR). Recently, TCTP has also been proposed to act as an indirect activator of mTOR. While it is known that mTOR plays a major role in the regulation of skeletal muscle mass, very little is known about the role and regulation of TCTP in this post-mitotic tissue. This study shows that muscle TCTP and mTOR signaling are upregulated in a range of mouse models (*mdx* mouse, mechanical load-induced hypertrophy, and denervation- and immobilization-induced atrophy). Furthermore, the increase in TCTP observed in the hypertrophic and atrophic conditions occurred, in part, via a rapamycin-sensitive mTOR-dependent mechanism. However, the overexpression of TCTP was not sufficient to activate mTOR signaling (or increase protein synthesis) and is thus unlikely to take part in a recently proposed positive feedback loop with mTOR. Nonetheless, TCTP overexpression was sufficient to induce muscle fiber hypertrophy. Finally, TCTP overexpression inhibited the promoter activity of the muscle-specific ubiquitin proteasome E3-ligase, MuRF1, suggesting that TCTP may play a role in inhibiting protein degradation. These findings provide novel data on the role and regulation of TCTP in skeletal muscle *in vivo*.

## INTRODUCTION

The highly conserved translationally controlled tumor protein (TCTP; a.k.a. p21, p23, Q23, and fortilin) was initially identified as a mitogen-stimulated protein whose mRNA was found in untranslated messenger ribonucleoprotein particles [1–5]. Since then, many studies have shown that TCTP is a regulator of cellular growth and proliferation, and that it is upregulated in a range of different cancer cell types [6–13]. Furthermore, the knockdown of TCTP has been shown to induce tumor reversion [14, 15]. Due to TCTP being a potential therapeutic target for the inhibition of cell growth [16, 17], it is important to gain a more comprehensive understanding of how TCTP protein expression is regulated, and of the molecular and cellular effects of increased TCTP in a range of different cell/tissue types.

TCTP protein expression was originally shown to be increased in serum stimulated cells in the presence of the transcription inhibitor, actinomycin D, thus establishing TCTP as a translationally regulated protein [3, 11]. Subsequent studies showed that elements within the 5′-untranslated region (5′-UTR) of the TCTP mRNA were responsible for repression of its translation under quiescent conditions [18]. Specifically, the 5′-UTR of TCTP mRNA was found to contain a tract of pyrimidines (5′-TOP) [19], a sequence that is found in the mRNAs of many mitogen-induced and translationally regulated ribosomal proteins, and in translation initiation and elongation factors (for review see [20]). Furthermore, modelling has shown that the 5′-UTR of the TCTP mRNA contains extensive secondary structure due to a very high content (~80%) of guanine (G) and cytosine (C) nucleotides [21], a characteristic that reduces translational efficiency under basal conditions, in part, by impeding ribosomal scanning and start codon recognition [22]. Combined, these data suggest that the control of TCTP protein expression is a highly regulated process that is stimulated under conditions that are associated with increased cellular growth.

A master regulator of cellular growth is the conserved serine/threonine protein kinase known as mTOR (the mammalian or mechanistic target of rapamycin) [23]. mTOR plays a major role in the regulation of mRNA translation (i.e., protein synthesis), with the potential to increase translational efficiency (i.e., translation initiation) and translational capacity (i.e., the number of ribosomes) [24]. Importantly, cell culture studies have shown that serum-stimulated increases in the expression of TCTP (and other proteins encoded by 5′-TOP mRNAs) are partially attenuated by the allosteric mTOR inhibitor, rapamycin [19, 25–27], indicating that TCTP is, in part, regulated in a rapamycin-sensitive mTOR-dependent manner.

In addition to TCTP being regulated by an mTOR-dependent mechanism, recent evidence has suggested that TCTP may in fact also act as an indirect activator of mTOR. Specifically, it has been reported in Drosophila, and in mammalian cells, that TCTP can act as a guanine exchange factor (GEF) for the small GTPase Rheb, a direct upstream activator of mTOR [13, 28, 29]. Indeed, knockdown of TCTP expression resulted in reduced cell size and this effect was associated with reduced mTOR signaling [13, 28]. It is important to note, however, that this has not been a universal observation, with some studies challenging this proposed mechanism [12, 30, 31]. Nonetheless, in support of this hypothesis, it was recently shown in cultured malignant peripheral nerve sheath tumor (MPNST) cells that TCTP overexpression was sufficient to activate mTOR signaling [32]. This finding prompted the authors to hypothesize the possibility of a positive feedback loop between mTOR and TCTP, at least in these mitotic tumor cells [32].

While there has been a significant amount of research into the role of TCTP in mitotic cells, very little is known about the role and regulation of TCTP in post-mitotic cells. Differentiated skeletal muscle fibers are post-mitotic cells that are able to increase (hypertrophy) or decrease (atrophy) their size via net changes in the rates of protein synthesis and protein degradation [33]. Furthermore, changes in mTOR signaling and protein synthesis have been implicated in the hypertrophic response of muscle to various growth-related stimuli [34]. For example, we have shown that mTOR kinase activity in skeletal muscle cells is necessary for mechanical loading-induced muscle hypertrophy and that the activation of mTOR signaling is sufficient to induce an increase in protein synthesis and muscle fiber hypertrophy [35–37]. To date, however, the molecular mechanism(s) that regulate mTOR signaling in skeletal muscle during various hypertrophic and atrophic conditions remain to be fully defined. Despite the mounting evidence that TCTP plays a role in the regulation of mTOR signaling and cell growth in mitotic cells, it currently remains to be determined whether TCTP is also upregulated during skeletal muscle growth via an mTOR-dependent mechanism, and whether an increase in TCTP is sufficient to activate mTOR, increase protein synthesis and induce muscle cell growth. In fact, very little is known at all about the role and regulation of TCTP in skeletal muscle cells.

Of the few studies to have reported on TCTP in skeletal muscle, one has shown that TCTP was one of several proteins upregulated in a mouse model of hypokalemic myopathy [38], a condition that displays signs of muscle fiber degeneration/regeneration [39]. Furthermore, TCTP has also been shown to be one of several proteins upregulated in the hyper-muscular mouse and bovine myostatin knockout models [40, 41]. This finding is of particular interest because myostatin signaling is known to inhibit muscle growth, in part, via the inhibition of mTOR and protein synthesis [42–44]. These studies suggest that TCTP may be upregulated in conditions associated with muscle remodeling and/or increased protein turnover, and that TCTP could potentially play a role in mTOR-mediated growth; however, more thorough mechanistic studies are required to begin to elucidate the function and regulation of TCTP in skeletal muscle. Therefore, the purpose of this study was to begin to investigate TCTP in mature skeletal muscle cells *in vivo*. More specifically, the aims of this study were to determine: 1) whether TCTP protein is upregulated, in an mTOR-dependent manner, during in skeletal muscle growth; 2) whether TCTP protein is correspondingly decreased during conditions that induce skeletal muscle atrophy; and 3) whether transient *in vivo* overexpression of TCTP is sufficient to activate mTOR signaling, increase protein synthesis and induce skeletal muscle fiber growth *in vivo*.

## RESULTS

### TCTP is upregulated in the *mdx* mouse model of Duchenne muscular dystrophy

**Figure 1:**
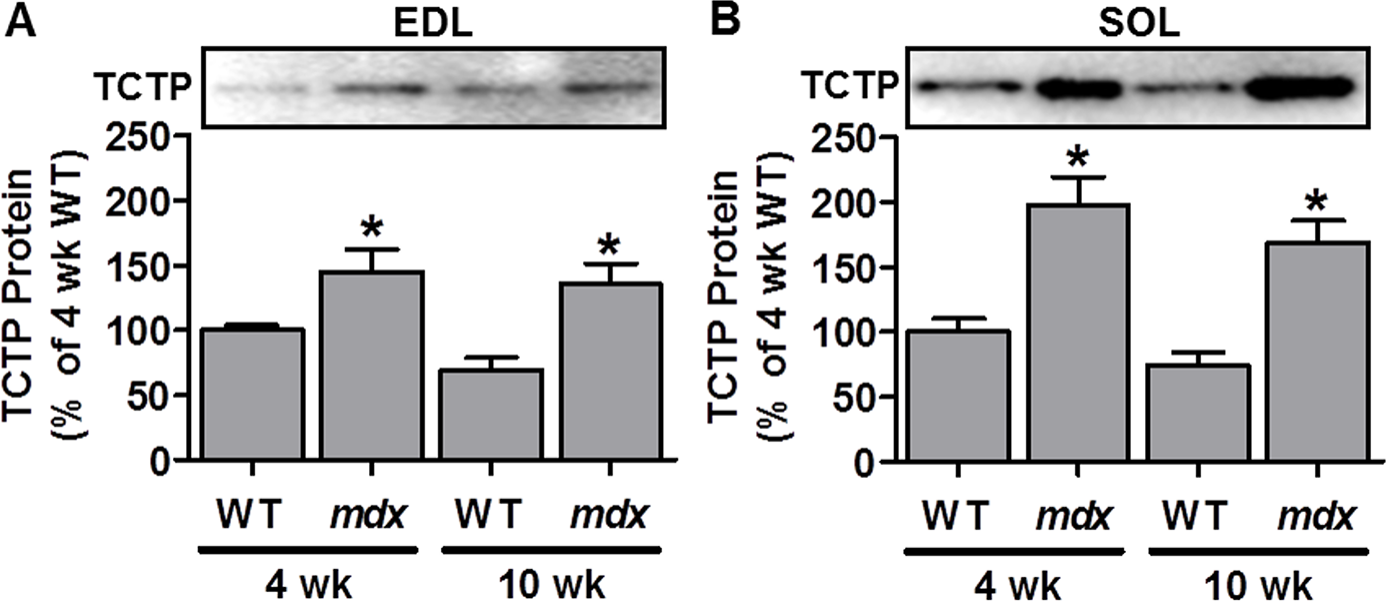
TCTP protein is elevated in dystrophic *mdx* mouse skeletal muscle Representative images and quantification of the Western blot analysis for TCTP in wild type (WT) and *mdx* extensor digitorum longus (EDL; **A**) and soleus (SOL; **B**) muscles at 4 and 10 wk of age. All values are expressed as a percentage of the values obtained from the 4 wk WT muscles. Values are reported as the mean + SEM, *n* = 5–9 / group. *Significantly different from WT muscles in the respective age group, *P* ≤ 0.05.

TCTP has previously been shown to be upregulated in a mouse model of hypokalemic myopathy, a condition that displays signs of muscle degeneration/regeneration [38, 39]. Another model that is characterized by distinct periods of muscle degeneration and regeneration is the *mdx* mouse model of Duchenne muscular dystrophy [45]. Specifically, *mdx* mice undergo an early period (3–4 weeks of age) of widespread muscle damage/necrosis followed by regeneration, and then an eventual stabilization period in adulthood (> 10 weeks) which is characterized by chronic, but low level, muscle fiber damage, regeneration and growth [45]. Therefore, we investigated whether TCTP may also be elevated in muscles from *mdx* mice. To this end, we examined TCTP protein in the fast-twitch extensor digitorum longus (EDL) and the slow-twitch soleus (SOL) hindlimb muscles of *mdx* mice at 4 and 10 weeks of age. As shown in Figure 1, TCTP was indeed elevated at these time points in both *mdx* muscles compared to controls. These findings show that, similar to the hypokalemic myopathy model [38], TCTP protein is upregulated in dystrophic *mdx* skeletal muscle. Furthermore, given that previous studies have shown elevated markers of mTOR signaling [46, 47] and increased rates of protein synthesis [48, 49] in *mdx* muscles, these data suggest that the increase in TCTP may be associated with increased mTOR signaling and with muscle remodeling.

### TCTP is upregulated during chronic mechanical overload-induced muscle growth via an mTOR kinase-dependent mechanism

**Figure 2:**
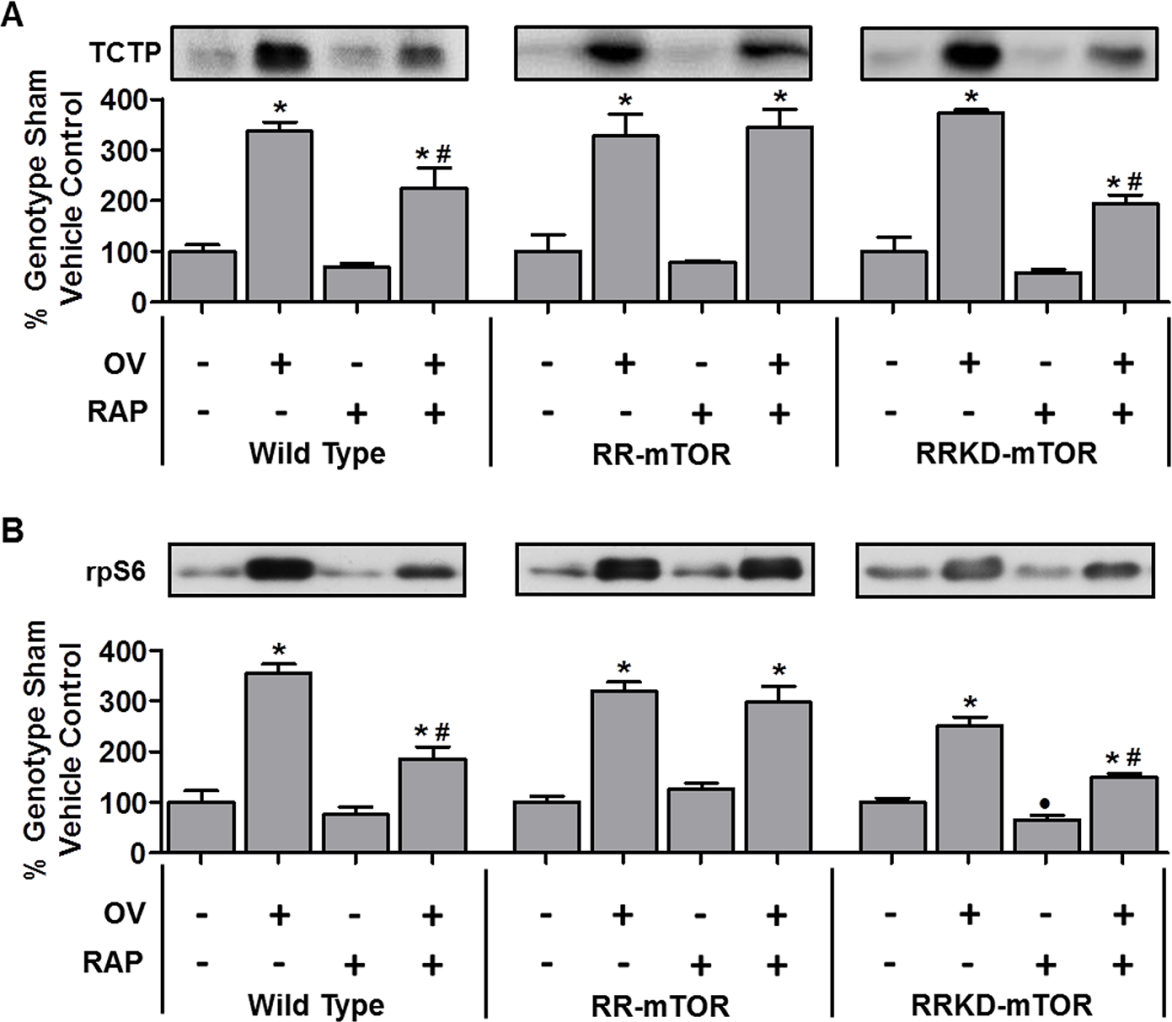
TCTP and rpS6 are upregulated during mechanical overload-induced muscle growth, in part, via a rapamycin-sensitive and mTOR kinase-dependent mechanism Plantaris muscles from wild-type, RR-mTOR, and RRKD-mTOR FVB/N mice were subjected to mechanical overload (OV+) or sham (OV–) surgeries. Following the surgery, the mice were administered a daily regime of either vehicle (RAP−) or 0.6 mg.kg^−1^ rapamycin (RAP+) injections. At 7 days post-surgery, the plantaris muscles were collected and subjected to Western blot analysis for TCTP and rpS6. For each genotype, TCTP and rpS6 were expressed as a percentage of the values obtained from the sham vehicle treated muscles. Values are reported as the mean + SEM, *n* = 3–12 / group. *Significant effect of OV within a given genotype and drug treatment, ·significant effect of RAP within the genotype-matched sham groups, ^#^significant effect of RAP within the genotype-matched SA groups, *P* ≤ 0.05.

To explore the potential mechanistic link between TCTP, mTOR signaling and muscle remodeling, we next examined whether mechanical overload (OV) would be associated with an increase in TCTP, and whether any increase in TCTP would occur in an mTOR kinase-dependent manner. Recently, using the mTOR inhibitor, rapamycin, combined with wild type (WT) mice, and mice that express rapamycin-resistant (RR-mTOR) and RR kinase dead (RRKD-mTOR) mutants of mTOR exclusively in skeletal muscle fibers, we demonstrated that mTOR is the rapamycin-sensitive element that confers OV-induced muscle fiber hypertrophy and that the kinase activity of mTOR is necessary for this event [35]. Therefore, using the same approach, TCTP was examined in plantaris muscles from WT, RR-mTOR and RRKD-mTOR mice that had been subjected to 7 days of OV [35], or a sham surgery, combined with daily rapamycin (0.6 mg.kg^−1^) or vehicle (DMSO) injections. We have previously demonstrated that this dose of rapamycin is sufficient to abolish OV-induced hypertrophy and the large (~22-fold) increase in 70 kDa ribosomal protein S6 kinase 1 Threonine 389 residue (p70^S6K1(Thr389)^) phosphorylation in muscles from WT and RRKD-mTOR, but not RR-mTOR, mice (see Figures 2 and 3 in ref [35]). As shown in Figure 2A, OV induced a marked increase in TCTP in muscles from vehicle-treated WT (3.4-fold), RR-mTOR (3.3-fold) and RRKD-mTOR (3.7-fold) mice compared to their respective sham vehicle controls. Furthermore, TCTP in muscles from OV and rapamycin-treated WT mice was ~33% lower compared to those from OV and vehicle treated mice (Figure 2A). Importantly, muscles from RR-mTOR, but not in RRKD-mTOR mice, were rescued from this inhibitory effect of rapamycin (Figure 2A). Together, these data show that TCTP is upregulated during OV-induced hypertrophy, in part, via a rapamycin-sensitive and mTOR kinase-dependent mechanism. However, despite the near complete inhibition of p70^S6K1(Thr389)^ phosphorylation in WT mice (see Figure 2 in [35]), rapamycin was unable to completely inhibit the OV-induced increase in TCTP. Thus, it can be concluded that the OV-induced increase in TCTP is mediated by both rapamycin-sensitive mTOR kinase-dependent and rapamycin-insensitive mechanisms.

**Figure 3:**
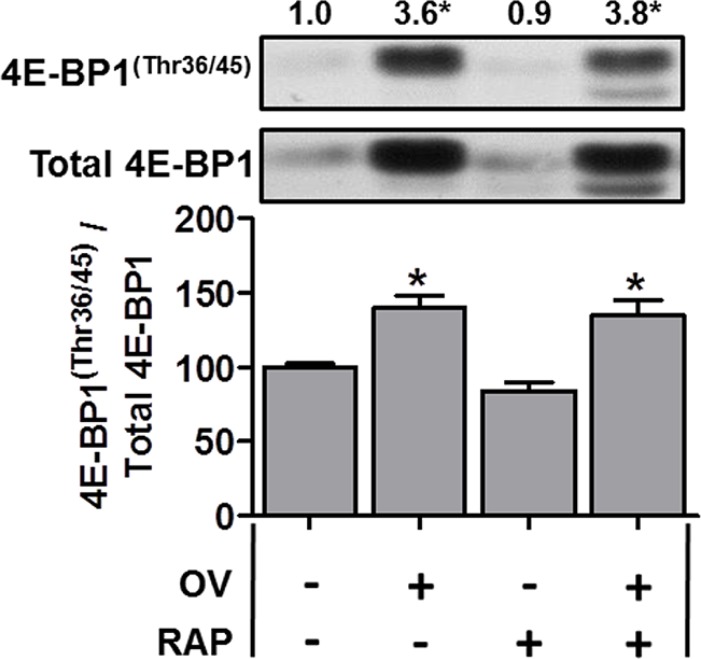
The mechanical overload-induced increase in 4E-BP1^(Thr36/45)^ phosphorylation is not inhibited by rapamycin Plantaris muscles from wild-type FVB/N mice were subjected to mechanical overload (OV+) or sham (OV−) surgeries. Following surgery, the mice were administered a daily regime of either vehicle (RAP−) or 0.6 mg.kg^−1^ rapamycin (RAP+) injections. At 7 days post-surgery, the plantaris muscles were collected and subjected to Western blot analysis for 4E-BP1^(Thr36/45)^ phosphorylation, and total 4E-BP1. All values are expressed relative to the values obtained from the sham vehicle treated muscles. Values in the graph are reported as the mean + SEM. The values at the top of the representative blot indicates the mean value for 4E-BP1^(Thr36/45)^ phosphorylation, *n* = 6–7/group. *Significant effect of OV within a given drug treatment, *P* ≤ 0.05.

To determine whether the partial inhibitory effect of rapamycin was a unique characteristic of the regulation of TCTP in OV muscles, we also examined the effect of OV, with or without rapamycin, on another 5′-TOP mRNA encoded protein, ribosomal protein S6 (rpS6, [20]). As shown in Figure 2B, OV induced a marked increase in rpS6 in muscles from WT, RR and RRKD vehicle-treated mice. Similar to TCTP, rapamycin only partially inhibited rpS6 expression in muscles from OV and rapamycin treated WT mice. Furthermore, the partial inhibitory effect of rapamycin on rpS6 expression was rescued in OV muscles from RR, but not RRKD, mice (Note: for examples of non-5′-TOP mRNA encoded proteins that are increased with SA but are insensitive to rapamycin (e.g., p70^S6K1^, Akt(PKB), FoxO3, AMPK and UBF), see Figure 5 in ref [35]). Combined, these data show that the OV-induced changes in the protein expression of the 5′-TOP mRNA-encoded TCTP and rpS6 are regulated by both rapamycin-sensitive mTOR kinase-dependent and rapamycin-insensitive mechanisms.

**Figure 4:**
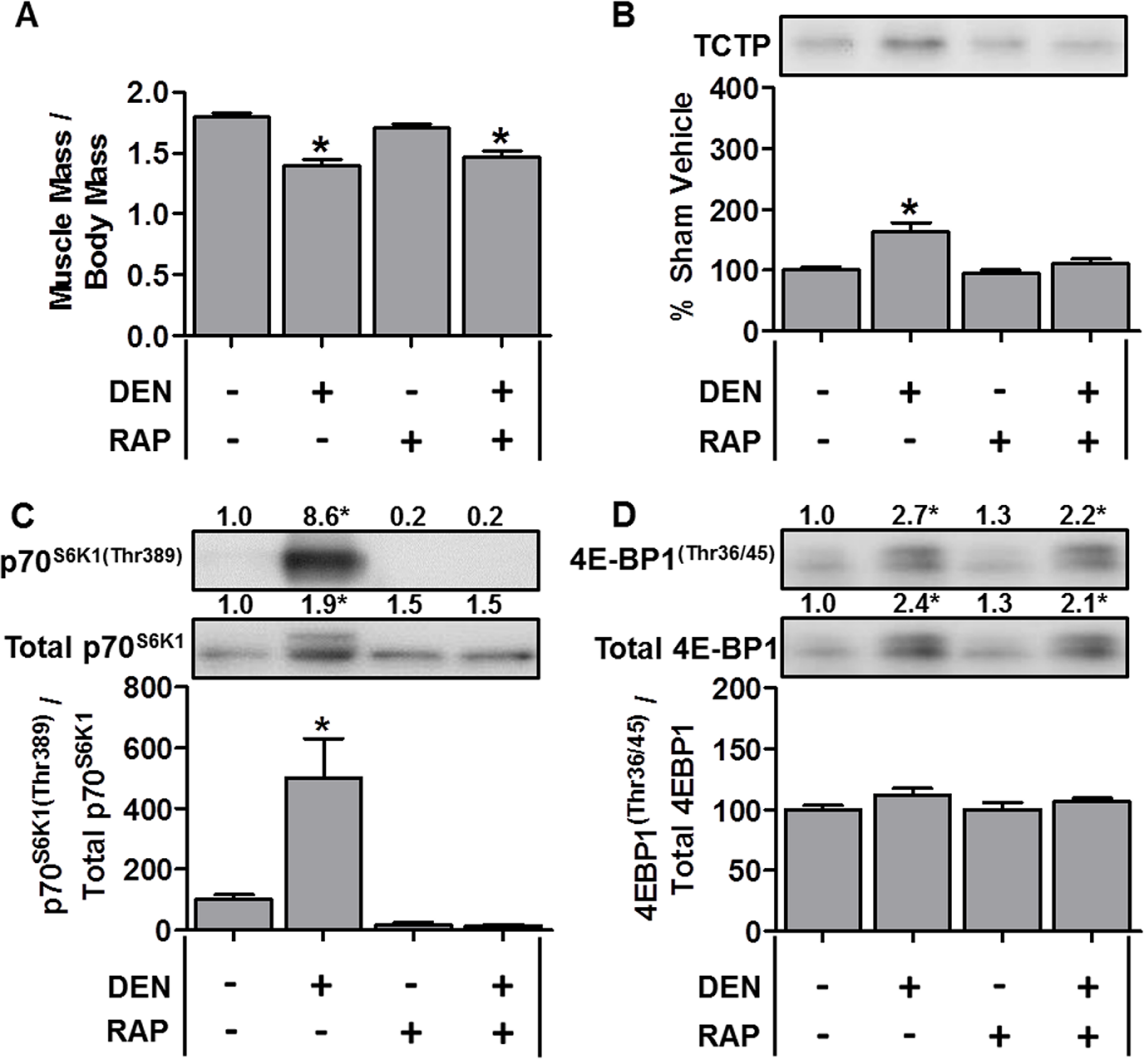
TCTP protein and mTOR signaling events are elevated during denervation-induced muscle atrophy Wild-type FVB/N mice were subjected to unilateral hindlimb denervation (DEN+) or a sham surgery (DEN–). Following the surgery, the mice were administered a daily regime of either vehicle (RAP−) or 1.5 mg.kg^−1^ rapamycin (RAP+) injections. At 7 days post-denervation, the tibialis anterior muscles were collected, (**A**) weighed, or (**B**–**D**) subjected to Western blot analysis with the indicated antibodies. All values are expressed as a percentage of the values obtained from the sham vehicle treated muscles. Values in the graphs are reported as the mean + SEM. The values at the top of the blots indicate the mean value for the respective phosphorylated and total proteins, *n* = 6–8/group. *Significant effect of DEN within a given drug treatment, *P* ≤ 0.05.

**Figure 5:**
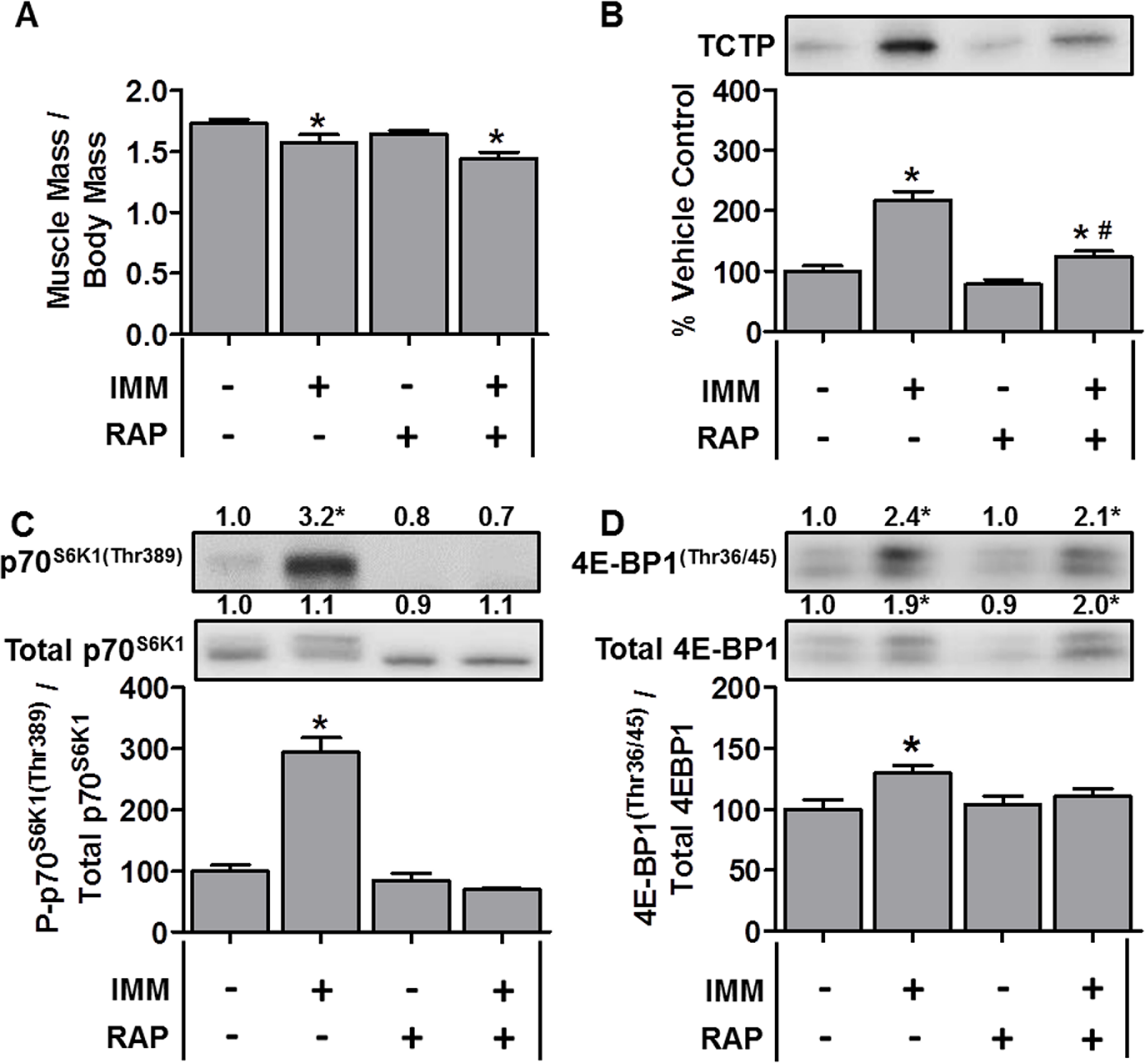
TCTP protein and mTOR signaling events are elevated during immobilization-induced muscle atrophy Wild-type FVB/N mice were subjected to unilateral hindlimb immobilization (IMM+) or allowed to move freely and administered a daily regime of either vehicle (RAP−) or 1.5 mg.kg^−1^ rapamycin (RAP+) injections. At 7 days post-immobilization, the tibialis anterior muscles were collected, (**A**) weighed, or (**B**–**D**) subjected to Western blot analysis with the indicated antibodies. All values are expressed as a percentage of the values obtained from the freely moving vehicle treated muscles. Values in the graphs are reported as the mean + SEM. The values at the top of the blots indicate the mean value for the respective phosphorylated and total proteins, *n* = 5–12/group. *Significant effect of IMM within a given drug treatment, *P* ≤ 0.05.

Recent evidence from non-muscle cell studies have shown that the expression of proteins encoded by 5′-TOP mRNAs (including TCTP) is largely regulated by mTOR-mediated phosphorylation of 4E-BP1 [25, 26]; however, it is also known that the function of 4E-BP1 is largely resistant to the inhibitory effect of rapamycin [50]. For example, the mTOR-mediated 4E-BP1^(Thr36/45)^ phosphorylation (4E-BP1^(Thr37/46)^ in humans) has been shown to be resistant to rapamycin, unlike the rapamycin-sensitive mTOR-mediated p70^S6K1(Thr389)^ phosphorylation (e.g., [50]). Thus, although our rapamycin treatment was sufficient to completely inhibit the overload-induced increase in p70^S6K1(Thr389)^ phosphorylation [35], it may not have been sufficient to fully inhibit 4E-BP1 phosphorylation, potentially leading to continued translation of TCTP despite the presence of rapamycin. Therefore, we next examined 4E-BP1 in muscles from WT mice subjected to OV. As shown in Figure 3, OV resulted in a marked increase in total 4E-BP1, 4E-BP1^(Thr36/45)^ phosphorylation (3.6-fold) and the phospho to total 4E-BP1 ratio (Figure 3); however, these parameters were unaffected by rapamycin treatment (Figure 3). Thus, in agreement with previous non-muscle studies, these data show that 4E-BP1^(Thr36/45)^ phosphorylation is resistant to the inhibitory effect of rapamycin, suggesting that continued mTOR-mediated phosphorylation of 4E-BP1 could play a role in facilitating the sustained translation of TCTP mRNA in OV muscles from mice that have been treated with rapamycin. When taken together, these results indicate that the expression of TCTP is regulated by a rapamycin-sensitive mTOR-dependent mechanism and by a rapamycin-insensitive mechanism that might involve the regulation of 4E-BP1 by mTOR.

### TCTP is upregulated in models of muscle atrophy

Given that TCTP was upregulated during OV-induced hypertrophy, we next explored whether TCTP would be altered under conditions that induce skeletal muscle atrophy. To this end, we first examined changes in TCTP in the hindlimb denervation model of muscle atrophy. For denervation experiments, mice were subjected to 7 days of unilateral hindlimb denervation via sciatic nerve transection. As shown in Figure 4A, denervation resulted in a ~22% decrease in tibialis anterior (TA) muscle mass; however, despite the decrease in muscle size, TCTP protein levels were modestly elevated (~1.6-fold, Figure 4B). We next examined whether this increase in TCTP was associated with an increase in mTOR signaling and found that denervation had indeed induced a marked increase in p70^S6K1(Thr389)^ phosphorylation (Figure 4C). Furthermore, while there was no change in the phospho to total 4E-BP1 ratio, the total amount of phosphorylated 4E-BP1^(Thr36/45)^ was significantly elevated (2.7-fold) in response to denervation (Figure 4D). To determine whether the denervation-induced increase in TCTP was mediated by mTOR signaling, we treated control and denervated mice with daily injections of rapamycin and found that rapamycin inhibited the increase in TCTP (Figure 4B). Furthermore, this inhibition of TCTP was associated with the abolition of the denervation-induced increase in p70^S6K1(Thr389)^ phosphorylation (Figure 4C); however, the total amount of 4E-BP1^(Thr36/45)^ phosphorylation remained markedly elevated (2.2-fold, Figure 4D). These data show that TCTP is not only upregulated in conditions that induce hypertrophy, but can also be upregulated, albeit to a lesser extent, under atrophic conditions. Furthermore, unlike what happened in response to OV, the modest denervation-induced increase in TCTP was completely inhibited by rapamycin.

To determine whether this increase in TCTP was somehow specific to atrophy induced by the loss of muscle innervation, we also examined TCTP during 7 days of hindlimb immobilization-induced atrophy [51]. As shown in Figure 5A, immobilization resulted in a 9.1% reduction in the TA muscle mass; however, despite this relatively small decrease in muscle size, TCTP was increased ~2.2-fold (Figure 5B). This increase in TCTP was associated with a marked increase in p70^S6K1(Thr389)^ phosphorylation, the total amount of phosphorylated 4E-BP1^(Thr36/45)^ and the phospho to total 4E-BP1 ratio (Figure 5C–5D). In contrast to denervation, however, rapamycin only partially inhibited the increase in TCTP (Figure 5B). This partial inhibition of TCTP was associated with the abolition of the immobilization-induced increase in p70^S6K1(Thr389)^ phosphorylation (Figure 5C); however, despite the ability of rapamycin to reduce the immobilization-induced increase in the 4E-BP1 phospho to total ratio, the absolute amount of phosphorylated 4E-BP1^(Thr36/45)^ remained significantly elevated (2.1-fold) (Figure 5D). Together, these data show that immobilization induced a larger increase in TCTP than denervation, and that this occurred via a rapamycin-sensitive and a rapamycin-insensitive mechanism(s).

### The overexpression of TCTP does not activate mTOR signaling

**Figure 6:**
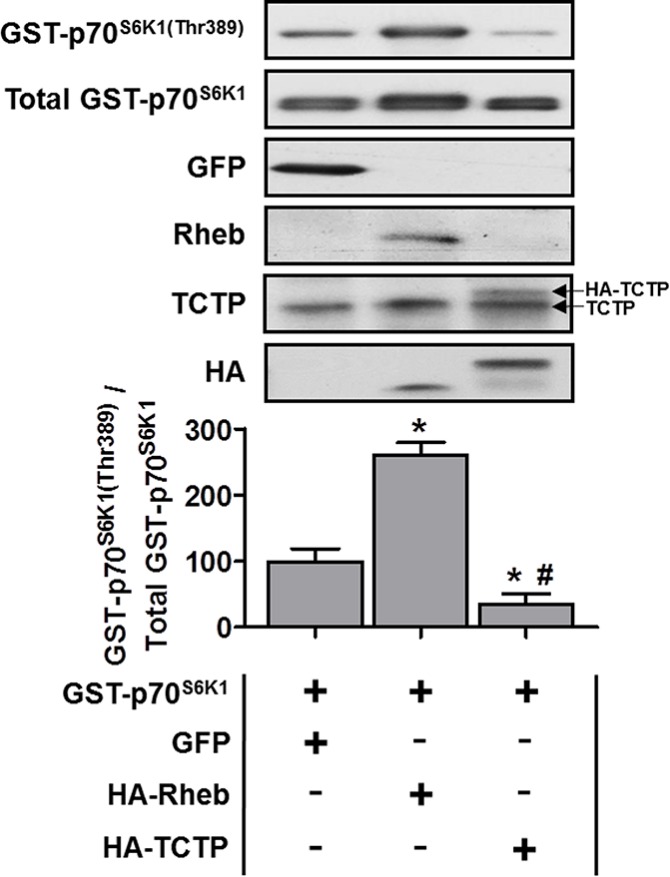
The effect of TCTP overexpression on mTOR signaling in skeletal muscle Wild type FVB/N mouse TA muscles were co-transfected with GST-tagged p70^S6K1^ (GST-p70^S6K1^) and GFP as a negative control, HA-tagged Rheb as a positive control or HA-tagged TCTP. At 3 days post-transfection, the muscles were collected and subjected to Western blot analysis with the indicated antibodies. The phosphorylated to total protein ratio for GST-P-p70^S6K1(Thr389)^ was calculated and expressed as a percentage of the values obtained in the GFP control samples. Arrows on the right hand side of the TCTP panel indicate bands representing endogenous TCTP and HA-tagged TCTP. Values are reported as the mean + SEM, *n* = 4 / group. *Significantly different from the values obtained in GFP transfected muscles, ^#^significantly different from the values obtained in Rheb transfected muscles. *P* ≤ 0.05.

Given that our data show that TCTP protein is upregulated, in part, by an mTOR-dependent mechanism, we next examined whether increased TCTP expression would, in turn, be sufficient to activate mTOR signaling. For these experiments, mouse TA muscles were co-transfected, via electroporation, with plasmid DNA encoding GST-tagged p70^S6K1^ (GST p70) [37, 44], and GFP (negative control), HA-tagged Rheb (positive control) or HA-tagged TCTP. After 3 days, muscles were collected and analyzed by Western blot for changes in the phosphorylation of GST P-p70^S6K1(Thr389)^ as a marker of mTOR signaling [37, 44]. As shown in Figure 6, the overexpression of Rheb induced a ~2.6 fold increase in GST-p70^S6K1(Thr389)^ phosphorylation compared to the GFP control; however, the overexpression of TCTP reduced GST-p70^S6K1(Thr389)^ phosphorylation to ~35% of the GFP control. These data indicate that an increase in TCTP protein levels actually inhibits mTOR signaling, and argues against the possibility of a positive feedback loop between mTOR and TCTP, in differentiated skeletal muscle cells.

### The overexpression of TCTP induces muscle fiber hypertrophy

**Figure 7:**
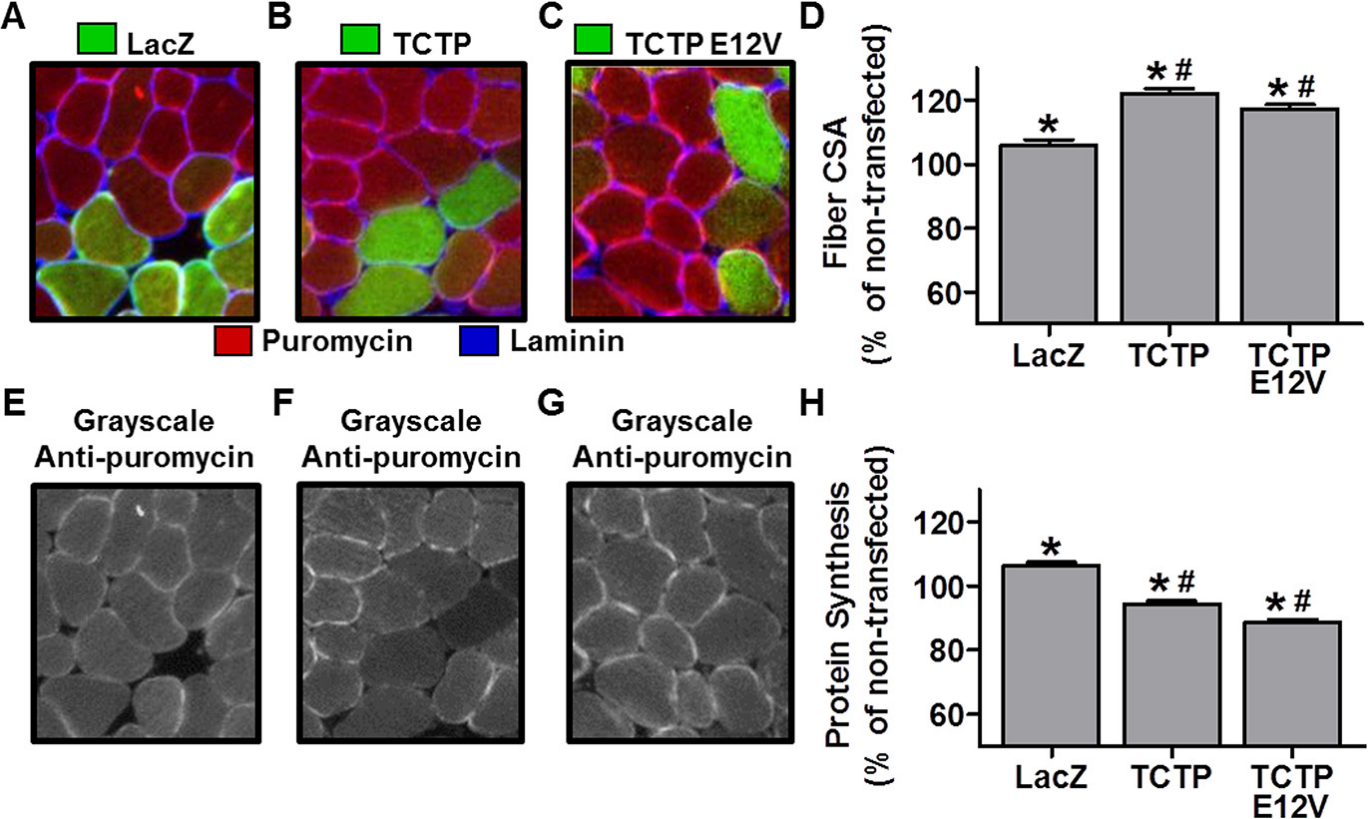
The effect of TCTP overexpression on muscle fiber cross-sectional area and rates of protein synthesis Wild type FVB/N mouse TA muscles were transfected with LacZ as a control or HA-tagged wild type TCTP or HA-tagged E12V TCTP mutant. At 7 days post-transfection, mice were injected with puromycin 30 min before the collection of the muscles. Muscles were then subjected to immunohistochemistry for LacZ or the HA tag (green), laminin (blue) and puromycin (red) (**A**–**C**) Representative merged images of anti-LacZ (A) or anti-HA (B, C), and anti-laminin and anti-puromycin signals. (**E**–**G**) Gray-scale images of the signal for puromycin shown in A, B and C, respectively. (**D**, **H**) Fiber cross-sectional area (CSA) (D) and puromycin staining intensity (i.e., protein synthesis) (H) in LacZ and HA-tagged wild type TCTP or E12V TCTP transfected fibers, expressed relative to the mean value obtained in non-transfected (control) fibers from the same section. Values are reported as the mean + SEM, *n* = 315-374 fibers/group from 4 independent samples/group. *Significantly different from the values obtained in non-transfected fibers. ^#^Significantly different from LacZ transfected fibers. *P* ≤ 0.05.

Although TCTP overexpression was not sufficient to activate mTOR signaling, it remained possible that TCTP could still induce growth in skeletal muscle. Therefore, we sought to determine whether the *in vivo* overexpression of TCTP would be sufficient to induce an increase in muscle fiber size. To this end, we used electroporation to transfect mouse TA muscles with plasmid DNA encoding HA-tagged TCTP or LacZ as a control condition. The muscles were collected at 7 days post-transfection for measurements of muscle fiber cross-sectional area (CSA). As shown in Figure 7A–7D, the CSA of TCTP transfected fibers was 22% larger than non-transfected fibers from the same sections, and 17% larger than control LacZ transfected fibers, indicating that TCTP is indeed sufficient to induce a hypertrophic response in skeletal muscle fibers *in vivo*.

To further test for a possible role of TCTP as a Rheb GEF, we also transfected muscles with a HA-tagged E12V mutant of TCTP, in which a critical glutamic acid residue (E12), located in TCTP's putative GTPase binding groove, was mutated to valine (V) [13, 28]. This mutation has been reported to abolish TCTP's GEF activity towards Rheb [13, 28]. Thus, if the wild type (WT) TCTP-induced hypertrophy was related to TCTP's putative interaction with Rheb, then overexpression of the E12V TCTP mutant protein should not induce an increase in fiber size. As shown in Figure 7C and 7D, however, the CSA of HA positive fibers was 17% larger than non-transfected fibers from the same sections and 12% larger than control LacZ fibers. Overall, these data indicate that TCTP is sufficient to induce skeletal muscle hypertrophy via a mechanism that does not require TCTP's reported Rheb GEF activity.

Muscle fiber size is ultimately determined by the net difference in rates of protein degradation and protein synthesis. Thus, we next wanted to determine whether TCTP-induced hypertrophy was associated with an increase in rates of protein synthesis. We therefore, examined the effect of WT and E12V TCTP overexpression on muscle fiber protein synthesis using our recently developed and validated immunohistochemical SUnSET technique which measures the rate of incorporation of the antibiotic puromycin into newly synthesized peptides [52]. For these experiments, WT TCTP and E12V TCTP were transfected into mouse TA muscles, and then the muscles were collected 30 min after an IP injection of puromycin at 7 days post-transfection [52]. Using this technique, we found that the rates of protein synthesis in WT and E12V TCTP transfected fibers were 11.1% and 16.6% lower than LacZ transfected control fibers (Figure 7F–7H). These data indicate that the TCTP-induced muscle fiber hypertrophy was not mediated by an increase in protein synthesis and therefore suggest that the hypertrophic effect of TCTP may instead be due to inhibition of protein degradation.

### TCTP overexpression inhibits the promoter activity of the muscle-specific E3 ligase, MuRF1

**Figure 8:**
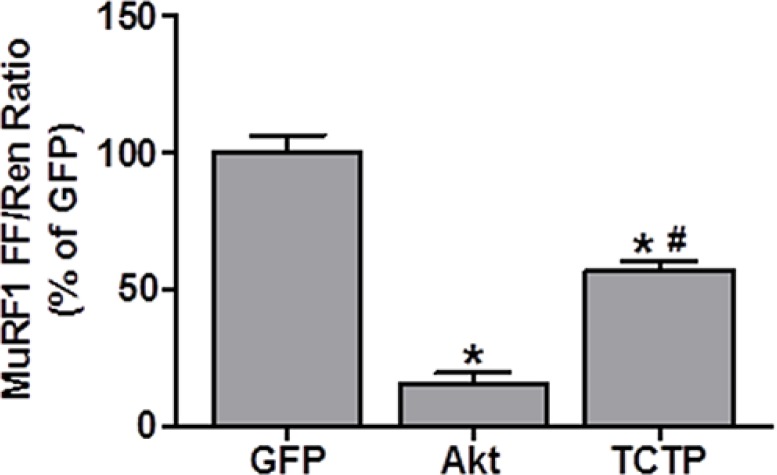
TCTP inhibits MuRF1 promoter activity Wild type FVB/N mouse TA muscles were co-transfected with GFP as a negative control, HA-tagged Akt as a positive control or HA-tagged TCTP, as well as the pRL-SV40 Renilla (Ren) luciferase reporter, and the MuRF1 promoter firefly (FF) luciferase reporter. At 3 days post-transfection, the muscles were collected and FF and Ren luciferase activities were measured with a dual-luciferase assay. Measurements of the relative light units produced by FF luciferase were normalized to that produced by Ren luciferase and this ratio was expressed as a percentage of the values obtained in the GFP transfected muscles. Values are reported as the mean + SEM, *n* = 4–5/group. *Significantly different from the GFP-transfected muscles. ^#^Significantly different from Akt-transfected muscles, *P <* 0.05.

Recent evidence suggests that TCTP may regulate protein degradation via the inhibition of caspase protease activity, autophagy and/or the UPS [9, 53–56]. As such, we wanted to see if we could find some evidence that might support a role for TCTP in inhibiting protein degradation. Unfortunately, unlike the SUnSET method for determining relative rates of protein synthesis, there are currently no equivalent methods that allow the direct determination of changes in protein degradation in single muscle fibers *in vivo*. Despite this, we reasoned that if TCTP overexpression was sufficient to inhibit protein degradation, this may result in a reduction in the activation of genes involved in this process. As such, we decided to examine whether TCTP would be sufficient to decrease the promoter activity of a muscle-specific UPS E3-ligase, Muscle RING-finger protein 1 (MuRF1). For these experiments, mouse TA muscles were co-transfected with GFP as a negative control, HA-tagged Akt (PKB) as a positive control [57] or HA-tagged TCTP, and a MuRF1 promoter luciferase reporter as previously described [44, 58]. As expected [57], the overexpression of Akt reduced MuRF1 promoter activity to ~15% of the GFP negative control, while the overexpression of TCTP reduced MuRF1 promoter activity by ~50% (Figure 8). This suggests the possibility that increased TCTP may indeed play a role in the inhibition protein degradation in skeletal muscle.

## DISCUSSION

This is the first study to investigate the role and regulation of TCTP in differentiated skeletal muscle cells *in vivo*. Our results show that TCTP is upregulated in a range of conditions (i.e., *mdx* mouse, mechanical load-induced muscle hypertrophy, and denervation- and immobilization-induced muscle atrophy) that involve some form of muscle remodeling. Furthermore, using transgenic mouse lines, and the mTOR inhibitor, rapamycin, we also showed the TCTP is increased under both hypertrophic and atrophic conditions via a mechanism that is, in part, mTOR-dependent. Our OV data indicate that, while part of the increase in TCTP is mediated by a rapamycin-sensitive mTOR-dependent mechanism, another component of the increase in TCTP is rapamycin-insensitive and may, in part, be regulated by a rapamycin-insensitive mTOR-dependent mechanism that involves 4E-BP1. We also show that, in skeletal muscle, TCTP is not sufficient to activate mTOR signaling and that TCTP is unlikely to play a role as a GEF for the Rheb GTPase or take part in the recently proposed positive feedback loop with mTOR [32]. Finally, we found that the overexpression of TCTP is sufficient to induce muscle fiber growth and this effect is not accompanied by an increase in protein synthesis, suggesting that the hypertrophic response may occur via the inhibition of UPS-mediated protein degradation.

In this study, we examined the regulation of TCTP during hypertrophic muscle growth induced by OV. Consistent with previous studies suggesting that TCTP is a growth-related protein [7, 27], we found that OV-induced muscle hypertrophy was associated with an increase in TCTP. Then, using rapamycin, and transgenic mice expressing RR- and RRKD-mTOR mutants, we demonstrated that the overload-induced increase in TCTP was, in part, rapamycin-sensitive and mTOR kinase-dependent. Furthermore, this partial rapamycin-induced inhibition of TCTP (and rpS6) was associated with the complete inhibition of the OV-induced increase in p70^S6K1(Thr389)^ phosphorylation [35] but not the inhibition of 4E-BP1^(Thr36/45)^ phosphorylation. This suggests that, under these growth conditions, the mTOR-mediated activation of p70^S6K1^ may have partially contributed to the rapamycin-sensitive component of the OV-induced increase in TCTP. Possible p70^S6K1^-mediated mechanisms that could play a role in the upregulation of proteins encoded by 5′-TOP mRNAs, such as TCTP [21, 59], include the inhibition of programmed cell death 4 (PDCD4)-mediated repression of the mRNA helicase, eIF4A, and/or by the phosphorylation of eIF4B which helps to recruit eIF4A to the translation initiation complex [59–61]. Another rapamycin-sensitive mTOR-mediated event that may have played a role in the up regulation of TCTP is eIF4G phosphorylation [62, 63], an event that has recently been proposed to play a role in 5′-TOP mRNA translation [64]. Regardless of the exact mechanism, the fact that rapamycin only partially inhibited the increase in TCTP (and rpS6) in OV muscles suggests that a rapamycin-resistant mechanism also plays a significant role in the regulation of 5′-TOP mRNA expression in hypertrophying skeletal muscle.

Recent non-muscle cell studies have shown that mTOR-mediated phosphorylation of 4E-BP1 plays a major role in promoting the translation of 5′-TOP mRNAs [25, 26] and that TCTP translation is regulated by eIF4E [27, 65], the direct binding target of 4E-BP1. Interestingly, studies have reported that the 4E-BP1 is largely resistant to the inhibitory effect of rapamycin [50]. In agreement, our results show that rapamycin had no effect on 4E-BP1^(Thr36/45)^ phosphorylation in OV muscles. This suggests that the increase in TCTP in OV muscles in the presence of rapamycin may, in part, be due to sustained mTOR-mediated 4E-BP1 phosphorylation and, thus, to continued cap-dependent translation of 5′-TOP mRNAs. This is supported by recent evidence showing that serum-stimulated increases in TCTP are inhibited to a greater extent by mTOR kinase inhibitors (e.g. PP242 and AZD8055), which inhibit both p70^S6K1^ and 4E-BP1 phosphorylation, than by rapamycin [27]. Overall, these data suggest that the upregulation of TCTP during mechanical overload-induced muscle growth occurs via both a rapamycin-insensitive mechanism (possibly mTOR-mediated phosphorylation of 4E-BP1) and a rapamycin-sensitive mTOR kinase dependent mechanism (e.g., p70^S6K1^ and/or eIF4G phosphorylation).

Interestingly, not only did SA increase 4E-BP1 phosphorylation, but it also resulted in a large increase in total 4E-BP1 protein (and to a lesser extent with denervation and immobilization). Increased 4E-BP1 protein has also been observed in various types of cancer, including prostate [66], breast [67], head and neck [68] and colorectal [69] cancers, and is often associated with poor prognosis (for recent reviews see [70, 71]). An increase in the abundance of this translational repressor in conditions associated with increased rates of protein synthesis seems counter intuitive and prompts the question of why? Previous studies have reported that 4E-BP1 gene expression is regulated by a range of transcription factors, including c-myc [72] and FoxO's [73, 74]. Importantly, we have shown that the protein abundance of c-myc [58], and FoxOs 1 and 3 [35], are increased by SA. Furthermore, FoxO activity is known to be elevated in atrophic conditions, such as immobilization and denervation [75–76]. Thus, an upregulation of 4E-BP1 protein in these models of muscle hypertrophy and atrophy may, in part, be due to an increased abundance and/or activity of c-myc and FoxO transcription factors. An alternative possibility is that the stability of 4E-BP1 protein may have been increased due to its hyperphosphorylation [70]. An increase in 4E-BP1 protein may serve to help maintain proteostasis by limiting the increase in the rate of translation with mechanical overload; however, a reason for the increase in 4E-BP1 in the atrophic conditions is less clear. Further studies will be needed to determine the role of an increase in 4E-BP1 protein during hypertrophic and atrophic conditions in skeletal muscle.

In addition to TCTP being upregulated during OV-induced muscle hypertrophy, we also found that TCTP was elevated in muscles undergoing atrophy. Moreover, this increase in TCTP was again associated with increased mTOR signaling and was inhibited to varying degrees by rapamycin. An increase in mTOR signaling in muscles undergoing atrophy is perhaps somewhat counter intuitive; however, this has been demonstrated previously in rodents [51, 77–80] and may, in part, be related to increased amino acid availability due to elevated rates of protein degradation [81]. When we compare the increase in TCTP between our atrophic and hypertrophic conditions, we find that the increase in TCTP in denervated and immobilized muscles was less than that found in the overloaded muscles (1.6- and 2.2- vs. 3.4-fold, respectively). This may be due to the relatively smaller increases in p70^S6K1^ and 4E-BP1 phosphorylation and/or to an accelerated turnover of TCTP due to the elevated global rates of protein degradation in these models [82–84]. Indeed, TCTP has recently been shown to be poly-ubiquitinated and degraded in a UPS-dependent manner [85]. An increase in TCTP degradation may also have contributed to TCTP appearing to be more sensitive to rapamycin, when compared to overloaded muscles. This may also help to explain, in part, the differences between the two atrophic conditions. For example, denervated muscles had a greater decrease in muscle mass compared to immobilized muscles (22% vs 9.1%), suggesting that protein degradation rates were higher in denervated muscles. Consistent with higher rates of protein degradation, denervated muscles also had a smaller increase in TCTP (1.6- vs 2.2-fold), which appeared to be more sensitive to the inhibitory effect of rapamycin. As a consequence, it is difficult to speculate on the relative contributions of rapamycin-sensitive vs. rapamycin-insensitive mechanisms to the upregulation of TCTP in these atrophic models. Nevertheless, our data clearly show that the up regulation of TCTP is mechanistically linked to mTOR activation under these two atrophic conditions. Overall, the implication of these results is that, although TCTP has often been described as a growth-related protein, the fact that TCTP was upregulated during hypertrophic and atrophic conditions suggests that, instead of being ‘growth-related’ *per se*, TCTP is upregulated in conditions that involve the activation of mTOR, regardless of whether the cell/tissue is undergoing an increase or decrease in size.

If an increase in TCTP protein is at least partially dependent on the activation of mTOR, then what possible mTOR-related function(s) could TCTP have within the cell? Several non-muscles studies have provided evidence that TCTP can act as a GEF for Rheb, a direct activator of mTOR signaling, and that TCTP is necessary for mTOR signaling [13, 28, 32, 86]. Furthermore, it has recently been reported that an increase in TCTP was sufficient to activate mTOR signaling, prompting the suggestion that TCTP might function in a positive feedback loop with mTOR [32]. However, contrary to this hypothesis, our data shows that, in skeletal muscle, an increase in TCTP expression is not sufficient to activate mTOR signaling. These results clearly argue against the existence of such a positive feedback loop in skeletal muscle cells. While we cannot rule out that TCTP may still play a necessary role in the activation of mTOR signaling in skeletal muscle, other recent non-muscle cell studies have also failed to find evidence to support this role [12, 30, 31]. Future muscle-specific knockdown or knockout studies will be needed to definitively determine whether TCTP is indeed necessary for the activation of mTOR in skeletal muscle.

Another interesting and unexpected finding of this study was that the overexpression of TCTP resulted in a decrease in protein synthesis. This finding, in part, may be related to the TCTP-induced inhibition of mTOR signaling; however, previous studies have shown that rapamycin-induced inhibition of mTOR signaling is not sufficient to inhibit basal rates of protein synthesis in skeletal muscle [87–89]. Another potential explanation relates to TCTP's previously proposed roles as an inhibitor of eukaryotic elongation factor 1B's (eEF1B) guanine nucleotide exchange factor (GEF) activity [90, 91] and as a guanosine nucleotide dissociation inhibitor (GDI) for eEF1A [92, 93]. In these roles, TCTP would inhibit the release of GDP from eEF1A which, in turn, would slow down the rate at which eEF1A binds GTP and aminoacyl-tRNAs, thus slowing the subsequent delivery of amino acids to the ribosome [94]. As such, an increase in TCTP would be predicted to slow translation elongation, and thus, inhibit the overall rate of protein synthesis. Although this proposed role for TCTP could be viewed as detrimental to cell survival or growth, recent evidence suggests that even a relatively minor slowing of peptide elongation may significantly reduce translation errors and nascent protein misfolding, leading to improved proteostasis [95–97]. In turn, this would result in a reduced need for the removal of error-containing or misfolded proteins via the UPS [95]. Hence, an increase in TCTP in models with increased mTOR signaling, such as OV, may play an important role in limiting the rate of translation to help minimize translational errors that accompany the increased rates of protein synthesis. Although attractive, more studies are required to further investigate this hypothesis.

One final question that arises from our study is how did TCTP overexpression induce an increase in muscle fiber size, despite a decrease in mTOR-signaling and protein synthesis? If muscle fiber size is ultimately determined by the net difference in the rates of protein synthesis and protein degradation, then our data suggests that TCTP overexpression may have induced a net decrease in protein degradation. Cellular proteins are degraded through several processes including via the activation of caspase proteases, autophagy and the UPS [98]. In this context, it is interesting to note that the overexpression of TCTP has been shown to inhibit caspase-3-like activity [53, 54], while the knockdown of TCTP can lead to an increase in caspase-3 activity [9]. Recent studies have also found an interaction between TCTP and the autophagy-related proteins ATG5, ATG12, and ATG16L1 [55]. Furthermore, the abundance of LC3-II, a marker of autophagy, was increased with TCTP knockdown under basal conditions [55], suggesting that TCTP may negatively regulate autophagy-mediated protein degradation. Finally, it has recently been reported that the yeast homologue of TCTP, Mmi1, acts as an inhibitor of the UPS [56]. In support of this putative role in inhibiting protein degradation, our results show that the overexpression of TCTP was sufficient to inhibit the promoter activity of the muscle-specific UPS E3-ligase, MuRF1. When combined with the proposed roles for TCTP as an eEF1A GDI or as an inhibitor of eEF1B GEF activity (see above), these data suggest that TCTP has the potential to inhibit cellular protein degradation. Further studies are, however, needed to more comprehensively explore this hypothesis.

## MATERIALS AND METHODS

### Animals

### Dystrophic *mdx* mice

Wild-type (C57/BL10ScSn) and *mdx* mice, 4 and 10 weeks old, were bred at the La Trobe Animal Research and Teaching Facility using breeding pairs obtained from the Animal Resource Centre (Western Australia, Australia). Mice were housed under a 12-h light/dark cycle with ad libitum access to food and water. Mice were killed by overdose of Nembutal (Sodium Pentobarbitone, ~10 ul.g^−1^) injection. Extensor digitorium longus (EDL) and soleus (SOL) muscles were rapidly excised and immediately snap frozen in liquid nitrogen and stored at -80°C until analysis by Western blotting. All procedures in this portion of the study were approved by the La Trobe University Animal Ethics Committee.

### FVB/N mice

Female FVB/N mice, 8–10 weeks old, were used for all other conditions. Mice were housed under a 12-h light/dark cycle with ad libitum access to food and water. FVB/N mice with human skeletal actin promoter driven expression of a FLAG tagged rapamycin-resistant (Ser2035Thr) mutant of mTOR (RR-mTOR), or a FLAG tagged rapamycin-resistant kinase dead (Ser2035Thr/Asp2357Glu) mutant of mTOR (RRKD-mTOR) have been previously described [35, 99]. These transgenic mice were bred with wild-type FVB/N mice and the offspring employed were either null (wild-type) or hemizygotes. Genotypes were confirmed with tail snips by PCR and 8–10 week old males were used for all experiments. Before all surgical and immobilization procedures, mice were anaesthetized with an intraperitoneal (IP) injection of ketamine (100 mg.kg^−1^) and xylazine (10 mg.kg^−1^). Muscles were rapidly excised and immediately snap frozen in liquid nitrogen and stored at −80°C for Western blotting analysis, or immediately submerged in optimal cutting temperature compound (Tissue-Tek; Sakura, Torrance, CA, USA) at resting length and frozen in liquid nitrogen chilled isopentane for immunohistochemical analysis. After tissue extraction, the mice were killed by cervical dislocation. All methods for this portion of the study were approved by the Institutional Animal Care and Use Committee of the University of Wisconsin-Madison.

### Mechanical overload

Mechanical overload (OV) was induced with synergist ablation surgeries in which the soleus and distal half of the gastrocnemius muscle were removed as previously described [35, 36]. Control mice were subjected to a sham surgery where an incision was made on the lower leg and then closed. Following the surgeries, incisions were closed with Vetbond surgical glue (Henry Schein, Melville, NY, USA). Mice were allowed to recover for 7 days and then the plantaris muscles were collected and subjected to Western blot analysis as described below.

### Denervation

Unilateral denervation surgeries were performed by making small incisions in the skin and underlying musculature on the lateral proximal thigh parallel with the femur. The sciatic nerve was then isolated and a 3–4 mm section of the nerve was cut out. Control mice were subjected to a sham surgery. Following the surgeries, incisions were closed with Vetbond surgical glue. Mice were allowed to recover for 7 days and then the TA muscle was collected and subjected to Western blot analysis as described below.

### Immobilization

Unilateral immobilization experiments were performed under anesthesia as recently described by You et al. [51]. This method involved fitting a custom made splint which immobilized the knee and ankle joints in extended and plantar flexed positions, respectively. Control mice were anesthetized but not placed in the splint. Mice were subjected to immobilization for 7 days and then the TA muscle was collected and subjected to Western blot analysis as described below.

### Rapamycin injections

Rapamycin was purchased from LC laboratories (Woburn, MA, USA) and was dissolved in DMSO to generate a 5 μg.μl^−1^ stock solution. The appropriate volume of the stock solution needed to inject mice was dissolved in 200 μl of phosphate-buffered saline (PBS). For the vehicle control condition, mice were injected with an equivalent amount of DMSO dissolved in 200 μl of PBS. Immediately following the OV, immobilization and denervation procedure, vehicle or rapamycin solutions were administered at the indicated dose via IP injections, and these injections were repeated every 24 h for 7 days.

### Western blotting

### Samples from *mdx* mice

Frozen sections from the EDL and SOL muscles were cut in a cryostat from the midpoint of the muscle (~30 × 10 μm 4 wk animals, ~20 × 10 μm 10 wk animals) and immediately placed into 1X SDS solution [3X SDS solution (0.125 M Tris-HCI, 10% glycerol, 4% SDS, 4 M urea, 10% mercaptoethanol and 0.001% bromophenol blue, pH 6.8) diluted 2:1 with 1× Tris.Cl (pH 6.8), 5 mM EGTA]. Western blotting was performed using a protocol similar to that described previously [100, 101]. A 4–5 point calibration curve was used to allow for comparison across gels [102, 103]. Briefly, an equal amount of protein from EDL and SOL samples and the calibration curve sample was separated on 4–15% gradient Criterion TGX Stain Free gels (BioRad, Hercules, CA). Total protein was imaged with a Stain Free Imager (BioRad). Proteins were then wet-transferred to nitrocellulose membrane. Membranes incubated in Pierce Miser solution (Pierce, Rockford, IL) and then blocked in 5% skim milk powder in 1% Tris-buffered, saline-Tween (TBST) for ~2 h at room temperature. Membranes were then incubated in primary antibodies overnight at 4°C and 2 h at room temperature, with primary antibody in 1% bovine serum albumin (BSA) in PBS with 0.025% Tween (PBST). The rabbit anti-TCTP (1:1000, #sc-30124) was obtained from Santa Cruz (Santa Cruz, CA, USA). Secondary antibody was goat anti-rabbit IgG, HRP conjugated, 1:60,000. Bands were visualized using West Femto chemiluminescent substrate (ThermoScientific, IL, USA) with images taken and densitometry performed using Image Lab software (v 5.1, BioRad). Total protein and specific protein densities were each expressed relative to their respective calibration curves and subsequently each protein was normalized to the total protein content [104]. All samples from wild-type and *mdx* mice were run in at least duplicate for each protein and averaged across gels.

### Samples from FVB/N mice

Frozen tissues were homogenized with a Polytron homogenizer (Kinematica AG, Lucerne, Switzerland) for 20 seconds in ice-cold WB buffer (40 mM Tris, pH 7.5; 1 mM EDTA; 5 mM EGTA; 0.5% Triton X-100; 25 mM β-glycerophosphate; 25 mM NaF; 1 mM Na3VO4; 10 μg/ml leupeptin; and 1 mM PMSF), and the whole homogenate was used for Western blot analysis. Sample protein concentrations were determined with a DC protein assay kit (Bio-Rad Laboratories, Hercules, CA, USA), and equivalent amounts of protein from each sample were dissolved in Laemmli buffer and subjected to electrophoretic separation on SDS-PAGE acrylamide gels. Following electrophoretic separation, proteins were transferred to a PVDF membrane, blocked with 5% powdered milk in Tris-buffered saline containing 0.1% Tween 20 (TBST) for 1 h followed by an overnight incubation at 4°C with primary antibody dissolved in TBST containing 1% BSA. The rabbit anti-total p70^S6K1^ (1:1000, 49D7, #2708), rabbit anti-Rheb (1:1000, #4935), rabbit anti-total 4E-BP1 (1:2000, 53H11, #9644), rabbit anti-phospho 4E-BP1^(Thr36/45)^ (1:1000, 236B4, #2855) and rabbit anti-GFP (1:1000, #2555) primary antibodies were obtained from Cell Signaling Technology (Danvers, MA). The rabbit anti-TCTP (1:3000, #sc-30124) and rabbit anti-phospho p70^S6K1(Thr389)^ (1:3000, #sc-11759) primary antibodies were obtained from Santa Cruz (Santa Cruz, CA, USA). The rat anti-hemagglutinin (HA, 1:2000, 1867431) primary antibody was obtained from Roche (Madison, WI, USA). After the overnight incubation, the membranes were washed for 30 min in TBST and then probed with a peroxidase-conjugated secondary antibody for 1 h at room temperature. Peroxidase-conjugated anti-rabbit and peroxidase-conjugated anti-rat antibodies were purchased from Vector Laboratories (Burlingame, CA, USA). Following 30 min of washing in TBST, the blots were developed on film, or with a Chemi410 camera mounted to a UVP Autochemi system (UVP, Upland, CA, USA), using regular enhanced chemiluminescence (ECL) reagent (Pierce, Rockford, IL, USA) or ECL Prime reagent (Amersham, Piscataway, NJ, USA). Once the appropriate image was captured, the membranes were stained with Coomassie Blue to verify equal loading in all lanes. Representative coomassie blue stains for Figures 2, 3, 4, 5 are shown in Supplementary Figure S1 (Note that in Figure 6, the signal for endogenous TCTP serves as the internal loading control). Densitometric measurements of the protein of interest were carried out using ImageJ (NIH; http://rsb.info.nih.gov/nih-image/).

### Plasmid constructs and purification

For all experiments, wild type (WT) TCTP and TCTP E12V overexpression was achieved by the *in vivo* transfection, or co-transfection, of a pHA-N3 (modification of the pEGFP-N3 plasmid; [28]) encoding HA tagged WT-TCTP or E12V TCTP plasmid construct (30 μg) (Kindly provided by Prof. Jianping Ding, Shanghai Institute of Materia Medica, China). LacZ (encoded by the pCMVβ plasmid; Marker Gene Technologies Inc., Eugene, OR, USA) or GFP (encoded by the pEGFP-C3 plasmid; Clontech, Mountain View, CA, USA) were co-transfected as control conditions. pRK5-myc-p70^S6K^-glutathione transferase (GST-p70^S6K^) was provided by Dr. Karyn Esser (University of Kentucky, Lexington, KY). pGL2-MuRF1-Luc (MuRF1 promoter luciferase reporter) was provided by Dr. Marco Sandri (University of Padova, Italy). pcDNA3.1-HA-Akt (HA-tagged wild type Akt) was provided by Dr. Jie Chen (University of Illinois at Urbana-Champaign, USA). pRL-SV40 Renilla Luciferase control plasmid was obtained from Promega (Madison, WI, USA). Plasmid DNA was amplified in DH5α Escherichia coli, purified with an EndoFree plasmid kit (Qiagen, Valencia, CA, USA), and resuspended in sterile PBS.

### *In Vivo* transfection via electroporation

Sterile plasmid DNA was transfected into mouse TA muscles by electroporation as described previously [37, 44]. In brief, mice were anesthetized, and a small incision was made through the skin covering the TA muscle. A 27-gauge needle was used to inject plasmid DNA solution into the proximal (6 μl) and distal (6 μl) ends of the muscle belly. After the injections, electric pulses were applied through 2 stainless steel pin electrodes (1-cm gap, Harvard Apparatus, Holliston, MA, USA) laid on top of the proximal and distal myotendinous junctions. Eight 20 ms square-wave electric pulses at a frequency of 1 Hz were delivered with an ECM 830 electroporation unit (BTX-Harvard Apparatus, Holliston, MA, USA) with a field strength of 160 V/cm. After the electroporation procedure, the incision was closed with Vetbond surgical glue. At 3 or 7 days after transfection, muscles were collected and subjected to Western blot or immunohistochemical analysis.

### Luciferase reporter assay

Plasmid DNA encoding the MuRF1 promoter reporter (20 μg) and the pRL-SV40 Renilla Luciferase control plasmid (1.25 μg), and plasmids encoding either WT-TCTP or GFP (30 μg), were co-transfected into TA muscles via electroporation. Muscles were collected at 3 days post-transfection and were homogenized with a Polytron homogenizer in passive lysis buffer (Promega, Madison, WI). Firefly and Renilla luciferase activities were measured with a FLUOstar Optima luminometer (BMG Labtech, Durham, NC) by using the Dual-Luciferase Reporter Assay kit (Promega) as described in the manufacturer's instructions. All firefly luciferase activities were normalized to the Renilla luciferase activity in the same sample.

### Immunohistochemical analysis of rates of protein synthesis and muscle fiber cross-sectional area

For *in vivo* measurements of protein synthesis using the SUnSET technique, mice were anesthetized at 7 days post-transfection and then given an IP injection of 0.04 μmol/g puromycin (Calbiochem, EMD Millipore, Billerica, MA) dissolved in 100 μl of PBS, as previously described [52, 105]. At exactly 30 min after the injection, TA muscles were excised and immediately submerged in optimal cutting temperature compound (Tissue-Tek; Sakura, Torrance, CA, USA) at resting length and frozen in liquid nitrogen chilled isopentane. Midbelly cross-sections (10 μm thick) were taken perpendicular to the long axis of the muscle with a cryostat and immediately fixed in −20°C acetone for 10 min. Sections were warmed to room temperature for 5 min and then incubated in PBS for 15 min, followed by a 1 h incubation in solution A [PBS with 0.5% BSA and 0.5% Triton X-100] containing anti-mouse IgG Fab (1:10; Jackson Immuno-Research). After three 5 min washes with PBS, samples were incubated for 1 h with solution A containing primary antibodies [mouse IgG2a Fc 2A monoclonal anti-puromycin (clone 12D10, 1:1000 (46)), rabbit IgG polyclonal anti-laminin (1:500, Sigma Aldrich, St Louis, MO, USA), and either chicken IgY polyclonal anti-LacZ (1:300, Abcam, Cambridge, MA, USA) or rat IgG1 monoclonal anti-HA (clone 3F10. 1:200, Roche, Madison, WI, USA)]. Sections were washed with PBS and then incubated for 1 h with solution A containing secondary antibodies [DyLight 594-conjugated anti-mouse IgG Fc 2a (1:500, Jackson Immuno-Research), Alexa 350-conjugated goat anti-rabbit IgG (1:1000, Invitrogen, Carlsbad, CA, USA), and either FITC-conjugated bovine anti-chicken IgY (1:100, Santa Cruz, Santa Cruz, CA, USA) or FITC-conjugated goat anti-rat IgG (1:100, Santa Cruz, Santa Cruz, CA, USA)]. Finally, the sections were washed with PBS and fluorescence images were captured with a Nikon DS-QiMc camera on a Nikon 80i epi-fluorescence microscope with TRITC (puromycin), FITC (LacZ or HA) and DAPI (laminin) cubes. The monochrome images were merged with Nikon NIS Elements D image analysis software. Measurements of the average cross-sectional area, and the average puromycin signal intensity, were obtained from randomly selected LacZ or HA-tagged WT TCTP/E12V TCTP transfected fibers, and an equal number of non-transfected fibers, by tracing the peripheral laminin stain. To calculate changes in fiber CSA and rates of protein synthesis, the CSA and intensity of the puromycin signal in LacZ and HA positive fibers was expressed relative to the mean CSA and intensity obtained in non-transfected fibers from the same muscle section, respectively. All analyses were performed by investigators that were blinded to the sample identification.

### Statistical analysis

All values are expressed as means (+SEM in graphs). Student's 2-tailed unpaired *t*-tests were used for all 2-group comparisons. Three group comparisons were performed using one-way ANOVA, followed by Student–Newman–Keuls post hoc analysis. Four group comparisons were performed using a two-way ANOVA followed by a Bonferoni post hoc analysis. Differences between groups were considered significant if *P <* 0.05. All data analysis was performed using GraphPad Prism 5.0 (GraphPad Software Inc., La Jolla, CA, USA).

## CONCLUSION

This is the first study to investigate the role and regulation of TCTP in skeletal muscle *in vivo*. The results show that TCTP protein is upregulated under both hypertrophic and atrophic conditions, in part, via an mTOR-dependent mechanism. Furthermore, this mTOR-dependent mechanism may consist of both rapamycin sensitive and rapamycin-insensitive components. The overexpression of TCTP, however, was not sufficient to activate mTOR signaling and is thus unlikely to play a role as a GEF for the Rheb GTPase in skeletal muscle, or to take part in a recently proposed positive feedback loop with mTOR. Nonetheless, TCTP overexpression was sufficient to induce muscle fiber growth in the absence of an increase in protein synthesis. Our preliminary results suggest that this hypertrophic response may occur, in part, via the inhibition of UPS-mediated protein degradation. Overall, these findings provide novel data on the role and regulation of TCTP in skeletal muscle cells.

## SUPPLEMENTARY MATERIALS FIGURES


