# Supplementary material for: Insights into the role and regulation of TCTP in skeletal muscle
Source: Oncotarget. 2016 Nov 1;8(12):18754–72. doi: 10.18632/oncotarget.13009 (PMC5386645; doi:10.18632/oncotarget.13009)
Supplement: Supplementary file 1 [file oncotarget-08-18754-s001.pdf]

Insights into the role and regulation of TCTP in skeletal muscle

Supplementary Materials

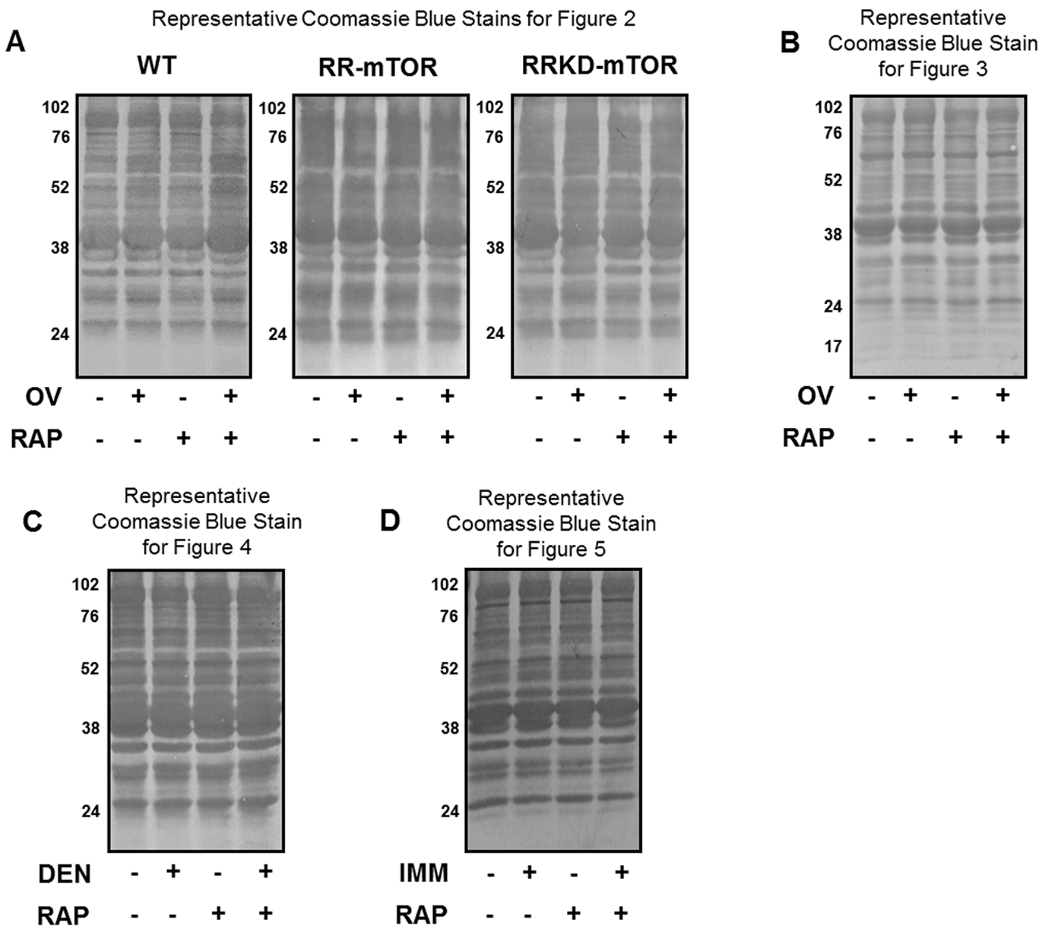

Supplementary Figure S1: Representative images of coomassie blue stained membranes. After Western blotting, membranes were stain with coomassie blue (CB) to verify equal loading of protein onto the gels. (**A**) Representative CB stains for Figure 2 which shows the analysis of muscles from WT, RR-mTOR and RRKD-mTOR mice subjected to mechanical overload (OV) with and without rapamycin (RAP) treatment. (**B**) Representative CB stain for Figure 3 which shows analysis of muscles from WT mice subjected to OV with and without RAP treatment. (**C**) Representative CB stain for Figure 4 which shows analysis of muscles from WT mice subjected to denervation (DEN) with or without RAP treatment. (**D**) Representative CB stain for Figure 5 which shows analysis of muscles from WT mice subjected to immobilization (IMM) with or without RAP treatment.
